# Non-disruptive uptake of anionic and cationic gold nanoparticles in neutral zwitterionic membranes

**DOI:** 10.1038/s41598-020-80953-3

**Published:** 2021-01-13

**Authors:** Ester Canepa, Sebastian Salassi, Federica Simonelli, Riccardo Ferrando, Ranieri Rolandi, Chiara Lambruschini, Fabio Canepa, Silvia Dante, Annalisa Relini, Giulia Rossi

**Affiliations:** 1 Department of Chemistry and Industrial Chemistry, University of Genoa, 16146 Genoa, Italy; 2 Department of Physics, University of Genoa, 16146 Genoa, Italy; 3 Materials Characterization Facility, Italian Institute of Technology, 16163 Genoa, Italy

**Keywords:** Nanoparticles, Computational biophysics, Membrane biophysics, Nanoscale biophysics, Membrane structure and assembly

## Abstract

The potential toxicity of ligand-protected nanoparticles (NPs) on biological targets is crucial for their clinical translation. A number of studies are aimed at investigating the molecular mechanisms shaping the interactions between synthetic NPs and neutral plasma membranes. The role played by the NP surface charge is still widely debated. We compare, via liposome leakage assays, the perturbation induced by the penetration of sub-6 nm anionic and cationic Au NPs into model neutral lipid membranes composed of the zwitterionic 1-palmitoyl-2-oleoyl-*sn*-glycero-3-phosphocholine (POPC). Our charged Au NPs are functionalized by a mixture of the apolar 1-octanethiol and a ω-charged thiol which is either the anionic 11-mercapto-1-undecanesulfonate or the cationic (11-mercaptoundecyl)-*N*,*N*,*N*-trimethylammonium. In both cases, the NP uptake in the bilayer is confirmed by quartz crystal microbalance investigations. Our leakage assays show that both negatively and positively charged Au NPs do not induce significant membrane damage on POPC liposomes when penetrating into the bilayer. By means of molecular dynamics simulations, we show that the energy barrier for membrane penetration is the same for both NPs. These results suggest that the sign of the NP surface charge, per se, does not imply different physicochemical mechanisms of interaction with zwitterionic lipid membranes.

## Introduction

Synthetic ligand-protected inorganic nanoparticles (NPs) have triggered important advances in different areas of nanomedicine, ranging from drug delivery and photothermal therapies to bioimaging and biosensing^1^. In particular, functionalized Au NPs have become one of the most widely studied NPs for biomedical applications^2,3^. Besides biocompatibility, this is due to the unique optical properties of gold which can be exploited both in diagnostic (in vitro sensing and in vivo imaging^4,5^) and in therapy (delivery applications^6–8^ and plasmonic therapies^8–10^). Some of these applications have already entered clinical trials^11,12^.

It is known that surface functionalization, together with NP size and shape, are key parameters to investigate and control NP cellular uptake, protein adsorption, and all other interactions with biological targets^13–16^. Yet a fully controlled design of synthetic NPs in terms of size and chemical composition is not trivial and remains a challenge^17^. Thiol-protected Au NPs are an excellent benchmark, as the available synthetic pathways allow for great control of the NP surface composition and size in comparison to other ligand-capped Au NPs. For this reason, thiolated Au NPs are ideal to investigate the basic and general principles of the interactions between synthetic NPs and biological interfaces, including model lipid membranes^13,18^. The advantages offered by gold surface functionalization with thiol self-assembled monolayers include limitation of NP core growth, protection from inter-particle aggregation, and long-term colloidal stability^19^. Thanks to the strong interaction between gold and sulfur, a vast library of thiol ligands (or mixtures thereof) can be exploited for surface functionalization^20^. The chemical nature of ligands defines most NP properties, including solubility, hydrophobicity and hydrophilicity, chemical reactivity, and eventually binding affinity to biointerfaces^21,22^.

The surface charge represents a crucial factor determining the behavior of functionalized NPs interacting with model lipid membranes. When looking at the interaction between charged Au NPs and model lipid membranes, it is tempting to interpret the experimental data by simple electrostatic arguments. Electrostatic attraction between oppositely charged NPs and bilayers certainly favors the formation of stable NP-lipid complexes. This is the case, for example, for positively and negatively charged metal or oxide NPs interacting with charged bilayers^23–25^. The interaction between oppositely charged NPs and lipid bilayers can cause transient damage to the membrane, as well. Liposome leakage assays by Goodman et al.^25^, for example, reported the disruptive effects of cationic NPs on negatively charged bilayers composed by a mixture of 1-stearoyl-2-oleoyl-*sn*-glycero-3-phospho-L-serine (SOPS) and 1-stearoyl-2-oleoyl-*sn*-glycero-3-phosphocholine (SOPC). Recently, combined experimental measurements and computer simulations have reported the favorable adsorption and aggregation, upon interaction, of cationic NPs on negatively charged model membranes^26,27^. Furthermore, perturbation of membrane composition and fluidity is shown, since negatively charged lipids cluster around cationic NPs^26^. The NP-membrane interaction is also associated with lipid extraction possibly causing membrane perturbation and destabilization^27^. The same reasoning is generally invoked to explain why, in vitro, cationic NPs are more toxic than anionic NPs to Gram-negative bacteria^28,29^. Nevertheless, electrostatic attraction is not a necessary ingredient to the formation of stable NP-bilayer complexes, nor to toxicity, which can take place also when the NP and the membrane have a Z-potential of the same sign^24,28,30^.

Even more subtle is the interpretation of the available experimental data on the interaction of charged NPs with the surface of membranes exposing neutral lipid headgroups, like in the extracellular leaflet of mammalian plasma membranes. Cationic and anionic Au NPs with a core in the 2–8 nm range can interact passively with mammalian cell membranes and model lipid bilayers^31–33^. The role played by the sign of the NP charge, though, is still debated. Neutron reflectometry studies by Tatur et al.^34^, suggest that anionic Au NPs could adhere to the surface of 1,2-distearoyl-*sn*-glycero-3-phosphocholine (DSPC) bilayer without penetrating it, at variance with cationic NPs that would interact with the zwitterionic DSPC membrane in a more disruptive way. According to Goodman et al.^25^, in pure SOPC bilayers, the membrane leakage induced by anionic NPs is larger than that of cationic NPs. On the contrary, Van Lehn et al.^32^, showed no membrane translocation of the fluorophore in multilamellar 1,2-dioleoyl-*sn*-glycero-3-phosphocholine (DOPC) vesicles in the presence of anionic Au NPs co-localized with the vesicle bilayers. Concerning computer simulations about NP-membrane interaction, there is a general consensus about the favorable interaction, spontaneously or by overcoming small free energy barriers, of anionic or cationic NPs with model neutral lipid bilayers^35–40^. Though, the role of the NP charge, at the molecular level, is again still unclear and debated.

In this paper, we aim at clarifying the role played by charged ligands during the interaction between monolayer-protected Au NPs and model neutral lipid bilayers. We want to disentangle the effects due to the NP charge from those due to other physical parameters, such as the NP size or the ligand length and flexibility. We thus consider sub-6 nm positively or negatively charged Au NPs, with a comparable core size dispersion and a controlled surface ligand composition. Both anionic (NP−) and cationic (NP+) Au NPs are functionalized by a mixture of two ligands: the apolar 1-octanethiol (OT) and a ω-functionalized alkyl thiol having the same chain length but a functional group with opposite charge. In particular, we used the anionic 11-mercapto-1-undecanesulfonate (MUS) for MUS:OT NP− and the cationic (11-mercaptoundecyl)-*N*,*N*,*N*-trimethylammonium (TMA) for TMA:OT NP+. These NP core and surface composition have become a reference for the study of NP-membrane interactions^25–27,34,40–43^, and many experimental results indicate the existence of a stable NP-membrane interaction with both ligand types, though a coherent molecular interpretation of the results is still lacking. We use a neutral membrane composed of the zwitterionic 1-palmitoyl-2-oleoyl-*sn*-glycero-3-phosphocholine (POPC). We tackle the NP-membrane interaction by means of a combined experimental and computational approach, relying on a close match between the models in silico and the experimental investigation. We quantify, using quartz crystal microbalance with dissipation monitoring (QCM-D) and fluorescence leakage assays, the extent of both NP− and NP+ membrane uptake and the resultant damage induced to the lipid bilayer. We find out that both NP− and NP+ stably interact with the bilayer in a non-disruptive way. We then offer a molecular-level interpretation of these results using coarse-grained molecular dynamics (MD) simulations^44^. Both NP− and NP+ interact favorably with the zwitterionic POPC bilayer sharing a common mechanism of interaction. The penetration mechanism mainly involves the one-by-one translocation of charged ligands through the hydrophobic core of the membrane. The associated free energy barrier turns out to be similar for both kinds of charged Au NPs.

## Results and discussion

### Experimental results: QCM-D and membrane leakage experiments

NP− and NP+ synthesis and characterization protocols are detailed in the “Methods” section and the “Supplementary Information”. From transmission electron microscopy (TEM) measurements, the gold core mean diameter was 2.7 ± 0.8 (σ) nm for MUS:OT NP− and 4.5 ± 1.1 (σ) nm for TMA:OT NP+ (see Supplementary Figure S1 and Supplementary Table S1). Dynamic light scattering (DLS) measurements yielded compatible hydrodynamic radii of 6.5 ± 0.2 nm for NP− and 7.7 ± 1.4 nm for NP+. The fraction of charged ligands was 80 ± 8% for NP− and 72 ± 11% for NP+, as determined by proton nuclear magnetic resonance (^1^H NMR) measurements after decomposition of the gold core. The NP Z-potential, measured in the experimental buffer (pH 7.4) in which the NP-membrane interaction took place, was − 31 ± 3 mV for NP− and + 25 ± 2 mV for NP+. These Z-potential values show that our NP dispersions retain a sufficiently high colloidal stability in the experimental conditions to avoid considerable NP aggregation. In all experiments, we added NP− and NP+ in the form of filtered aqueous dispersions (see “Methods”).

**Figure 1:**
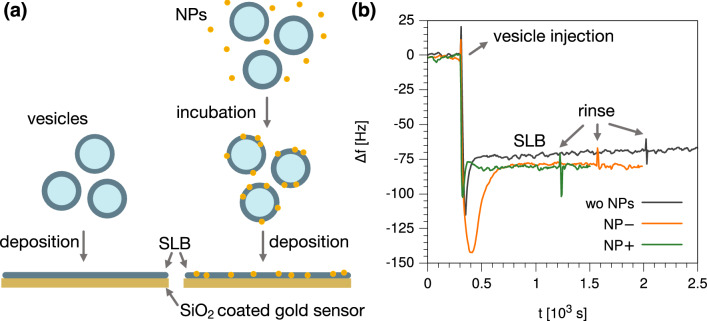
QCM-D quantification of NP− and NP+ uptake in POPC membranes. (**a**) Schematic drawing of sample preparation for QCM-D investigation. POPC vesicles and NP (NP− or NP+) were incubated and allowed to interact before vesicle fusion on the sensor. (**b**) Frequency change (3rd overtone), ∆*f*, recorded after injection in the QCM-D chamber (t = 300 s) of POPC vesicles (black curve), POPC vesicles incubated with NP− (orange curve), and POPC vesicles incubated with NP+ (green curve). The traces clearly indicate that the SLB formation occurs via vesicle fusion with different kinetics depending on the charge of the system, as often reported in the literature for anionic and cationic vesicles^48,49^. SLB formation took place within 600 s after vesicle adsorption and their subsequent fusion on the sensor for POPC and POPC/NP−, whereas for POPC/NP+ a direct SLB formation occurred. The frequency shift ∆*f* coincides for POPC vesicles incubated with NP− and NP+, and it is larger than that of POPC vesicles alone, indicating increased mass adsorption on the sensor.

Within our experimental time scales, extensive NP incorporation in neutral zwitterionic membranes has already been reported for charged AuNPs of similar core size and ligand molar ratio^27,32,34,45,46^. To quantify the uptake of NP− and NP+ in the POPC membrane, we performed QCM-D investigations using SiO_2_ coated gold sensors (see “Methods” for experimental details). It is known that POPC vesicles rapidly tend to adsorb and fuse onto SiO_2_ to form an essentially defect-free supported lipid bilayer (SLB) completely covering the sensor surface^47^. As shown in Fig. 1a, we incubated NP− or NP+ with POPC vesicles in the buffer before insertion into the QCM chamber. QCM results are reported in Fig. 1b (∆*f*) and Supplementary Figure S3 (dissipation changes); the frequency shift ∆*f* relative to the 3rd overtone is reported for POPC vesicles alone and POPC vesicles incubated with either NP− (POPC/NP−) or NP+ (POPC/NP+). When SLBs were formed, the measured frequency shifts, normalized by overtone number, were − 19.3 ± 2.0 Hz for POPC, − 26.9 ± 1.9 Hz for POPC/NP− and − 25.0 ± 2.4 Hz for POPC/NP+; these values correspond to an adsorbed mass ∆*m* (Eq. (1) in “Methods”) of 343 ± 35 ng/cm^2^ for the pure lipid vesicles, and to similar masses of 479 ± 34 ng/cm^2^ and 445 ± 43 ng/cm^2^ for the vesicles incubated with NP− and NP+, respectively. The larger masses found in the experiments with NPs clearly confirm the uptake of NP− and NP+ in the zwitterionic bilayer during incubation. As shown in Fig. 1b, the final mass values did not change after rinsing, indicating that all SLBs are stable and those with NPs stably retain their NP content.

**Figure 2:**
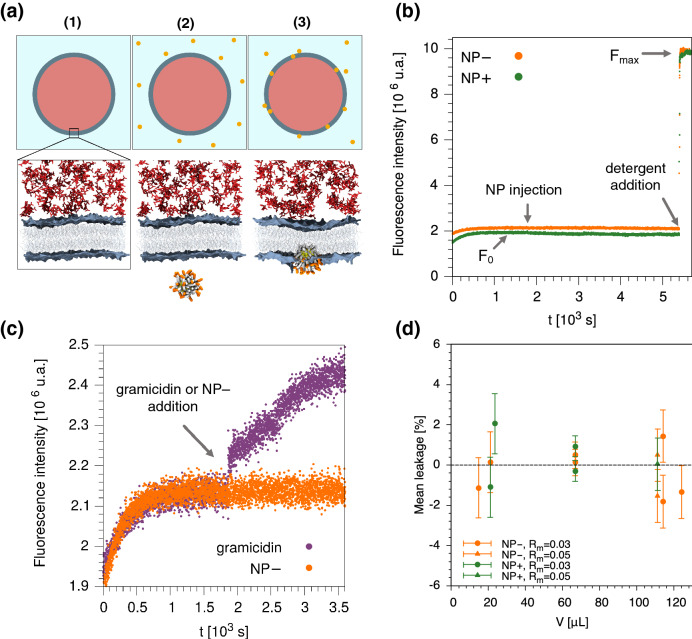
Leakage experiments on NP-vesicle suspensions. (**a**) Schematic drawing illustrating the three steps of our dye-leakage assays on calcein-loaded POPC liposomes: (1) liposomes before NP addition; (2) NP injection, (3) liposomes during the NP uptake. Simulation snapshots of a membrane portion are reported in the second row; membrane lipid heads are shown in blue (surface representation) and lipid tails in light-gray, calcein molecules in red, charged groups of the NP ligands in orange, and the rest of the NP in white (ligands) and yellow (Au core). (**b**) Typical leakage traces recorded before and after the addition of NP− (orange curve) and NP+ (green curve) to POPC liposomes. NP− and NP+ were added after the first 1800s, as explained in detail in “Methods”. *F*_*0*_ is the fluorescence level immediately before NP addition, while *F*_*max*_ is the final fluorescence level after the detergent-induced liposome rupture. In general, for both positively and negatively charged Au NPs, no content release (i.e. fluorescence intensity increase) was recorded during the NP-membrane interaction. In this specific experiment, NP at *R*_*m*_ = *m*_NP_/*m*_lip_ = 0.03 were added (V_NP−_ = 14.6 μL, V_NP+_ = 23.4 μL). (**c**) First 3600 s of the curve in (**b**) compared to the leakage-inducing effect of a low amount of gramicidin (250 nM). (**d**) Normalized fluorescence intensity of calcein (i.e. mean leakage %) as a function of the NP added volume. NP− (orange) and NP+ (green) at *R*_*m*_ = 0.03 (●) and 0.05 (▲) were tested. The mean leakage % was calculated over the last 10 min before the addition of the detergent.

After checking the uptake of NP− and NP+ in the POPC bilayer, we tested their ability to destabilize the lipid membrane by dye-leakage assays on POPC vesicles loaded with a self-quenched calcein solution (175 mM). We used small sonicated vesicles with Z-potential, measured in the experimental buffer, equal to − 5.5 ± 0.4 mV. This value, although slightly negative, can still be considered as characteristic of a neutral membrane^50^. All details on the experimental set-up are described in the “Methods” section, while vesicle characterization results are shown in Supplementary Table S1. Our dye-leakage assays on POPC vesicles can be divided into three steps, as described in Fig. 2a. Typical membrane leakage curves recorded before and after the addition of NP− and NP+ (NP/lipid mass ratio, *R*_*m*_ = *m*_NP_/*m*_lip_ = 0.03) are reported in Fig. 2b. Calcein fluorescence was monitored as a function of time and the fluorescence level did not change significantly after the injection of both NP− and NP+. The portion of the curve before and immediately after NP addition is enlarged in Fig. 2c and compared with a control experiment in which a low amount (250 nM) of the pore-forming peptide gramicidin^51^ was added to POPC liposomes (for the sake of clarity, only the NP− case is reported). The membrane permeabilization induced by gramicidin corresponds to a fast, small but clearly detectable fluorescence increase, indicating that our liposome system was sensitive to membrane permeability changes. As shown in Fig. 2b, calcein fluorescence intensity remains stable over time after the injection of NP− and NP+, suggesting that no membrane permeabilization occurred. The addition of detergent (sodium cholate) at the end of the experiment caused a fast liposome rupture with the complete release of the fluorescent dye. The instantaneous large fluorescence change recorded after the detergent-induced liposome rupture confirms that vesicles still retained their contents after NP addition. The final fluorescence level (*F*_*max*_, Fig. 2b) was used to normalize the leakage data, as described in Eq. (2) of the “Methods” section. Experiments were repeated increasing the NP concentration to *R*_*m*_ = 0.05, but no changes were recorded in fluorescence intensity. All leakage results are summarized in Fig. 2d, which reports the mean normalized fluorescence intensity of calcein recorded 1 h after the addition of NP− and NP+.

Oscillation around zero of the values shown in Fig. 2d is due to minor fluctuations in fluorescence intensity recorded after the addition of charged Au NPs. To understand whether the origin of these fluctuations could be due to the membrane uptake of charged Au NPs, we performed control experiments adding similar volumes of water aliquots (without NPs) and we observed that the effect of the water alone is comparable with those of both NP− and NP+ (see Supplementary Figure S4a). This confirmed that no minor leakage effect could be ascribable to the NP-membrane interaction. We performed additional leakage experiments on larger POPC liposomes obtained by extrusion (vesicle characterization is reported in Supplementary Table S1). Also in this case, no membrane leakage was induced by the addition of NP− and NP+ at *R*_*m*_ = 0.03 and 0.05 (see Supplementary Figure S4b). Our leakage results indicate that POPC membrane destabilization subsequent the interaction with charged Au NPs is negligible for both NP− and NP+, thus suggesting that if defects are formed in the lipid bilayer during NP uptake, they are transient and do not allow a significant leakage of the inner dye.

### Computational results: MD simulations

We tested the results of our QCM-D and dye-leakage experiments using coarse-grained MD simulations. As better detailed in the “Methods” section, we used model MUS:OT NP− and TMA:OT NP+ with 2 nm core size. We relied on the polarizable version of the Martini coarse-grained model that explicitly takes into account water orientational polarizability. This model is quantitatively reliable at the estimation of the energy barriers that characterize the NP-membrane interaction^52–54^. We simulated the NP-membrane interaction and sampled the free energy surface of the NP-membrane complex by means of unbiased MD and biased metadynamics^55^ calculations.

**Figure 3:**
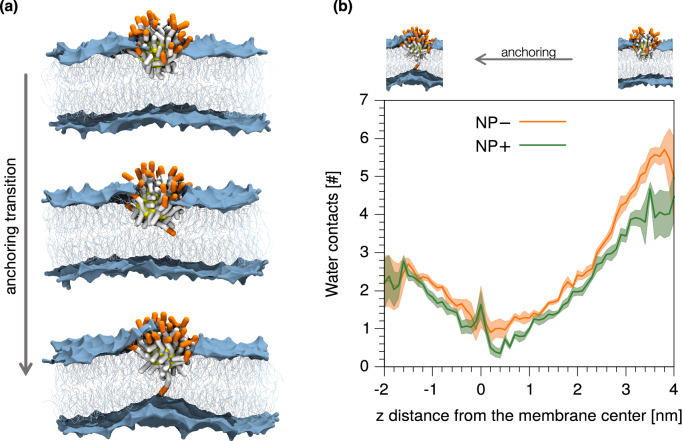
NP− and NP+ share the same mechanism of interaction with the membrane. (**a**) A charged ligand (orange beads represent the anionic terminal groups) makes the anchoring transition. Color code as in Fig. 2a. The same mechanism of interaction takes place with charged ligands showing cationic terminal groups. (**b**) The time average number of contacts between the biased ligand terminal and coarse-grained water beads as a function of the *z* distance between the terminal and the COM of POPC bilayer. The shaded area corresponds to the standard deviation associated to the time average.

NP− and NP+ share the same mechanism of interaction with the membrane. Our simulations show that the interaction of charged Au NPs with lipid bilayers is a process that involves the transition between different metastable states^40^. One transition, in particular, is interesting for our comparison between NP− and NP+. This transition, which we refer to as the “anchoring transition” and is shown in Fig. 3a, requires that the charged ligands of the NP, initially bound to the headgroup region of the entrance leaflet, cross the hydrophobic membrane core to bind to the distal leaflet. We previously showed that, for NP− with a patched surface ligand arrangement^54^, the charged ligand translocation can involve significant membrane deformation and transient membrane poration. Here we compare this step of the NP-membrane interaction for NP− and NP+ with a random surface arrangement of charged and hydrophobic ligands. As the transition requires the charged ligands to overcome a significant free energy barrier, the process cannot be observed during unbiased MD runs. We thus use metadynamics^55^ to accelerate the transition. In this set up, the dynamics of a single charged terminal of one ligand is biased along the reaction coordinate, which is the *z* component of the distance between the charged group and the center of mass (COM) of the membrane, to favor the ligand translocation across the membrane. More details on the metadynamics^55^ set up are reported in the “Methods” section. The visual inspection of the biased trajectories suggests that both the negatively and the positively charged ligand translocations induce significant membrane deformations, as shown in Supplementary Figure S5. For NP−, in six out of eight translocation processes we observed at least one water bead being transferred across the membrane together with the anchoring ligand. For NP+, the same happened in eight out of eight transitions. Figure 3b shows the average number of contacts between the charged terminal of the biased ligand and water, as a function of the reaction coordinate. When the charged ligand terminals approach the COM of the membrane, they are hydrated and the number of water beads surrounding the ligand is similar for NP− and NP+.

**Figure 4:**
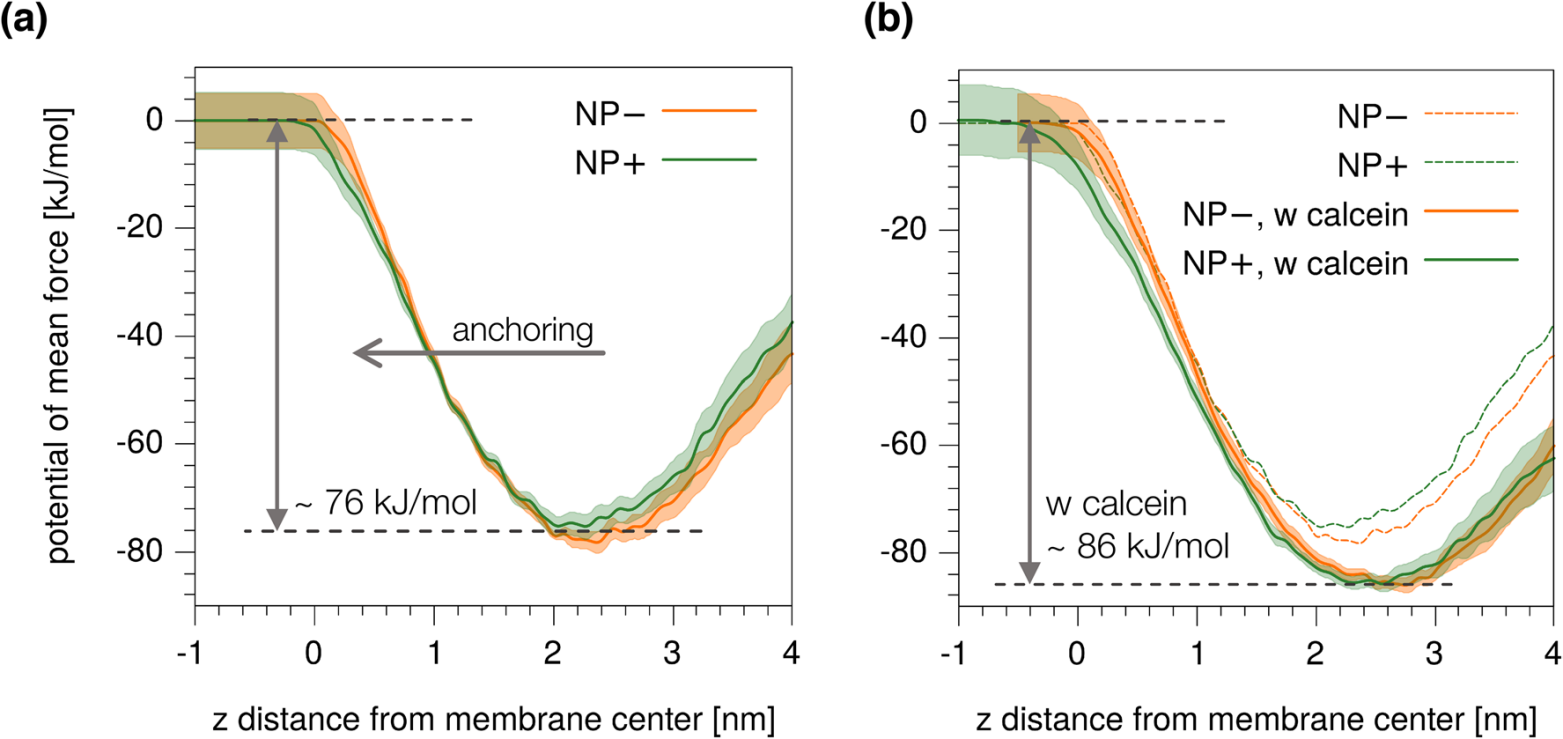
Potential of mean force for the translocation of a single charged ligand, bound to the NP, from the entrance to the distal leaflet. On the x*-*axis, we report the collective variable of the metadynamics runs, which is the *z* component of the distance between the charged terminal of the biased ligand and the centre of mass of the membrane. (**a**) Without calcein. (**b**) Comparison with and without calcein. The shaded area corresponds to the standard error; in (**b**) are reported only the error for the case with calcein only, for readability. See Ref.^54^ for details on the standard error estimation.

More quantitatively, we used metadynamics^55^ to calculate the free energy barriers for the translocation of the negatively and positively charged ligands (bound to the NP as in Fig. 3a) across the membrane^54^. Figure 4a shows the potential of mean force obtained for NP− and NP+. Again, no significant difference is observed for the anchoring barriers of the oppositely charged Au NPs, which set to 76 ± 6 kJ/mol for NP− and 77 ± 5 kJ/mol for NP+. NP− and NP+ thus share the same molecular mechanisms and energetics of interaction with the POPC bilayer. The presence of calcein within the liposome does not affect the mechanism and the energetics of NP-membrane interaction. We developed a Martini model of the calcein dye^56^, as shown in Supplementary Figure S6. We then set-up a system consisting of two parallel bilayers and two distinct water chambers between them. In one water chamber, we dissolved calcein molecules with a concentration of 175 mM, in accordance with the experimental set-up of leakage assays. Ions and counterions were added to the solution as described in the “Methods” section. We monitored the structural properties of the bilayers, such as the area per lipid and the density profiles of lipid heads and tails along the normal to the bilayer (see Supplementary Figure S7) and found no significant change of membrane properties with respect to the bilayer in absence of calcein. Then, one NP− (or NP+) was incorporated into one leaflet of a bilayer, choosing as entrance leaflet the one facing the pure water chamber. Again, we looked at the ligand anchoring transition via metadynamics^55^. As for the anchoring transition, calcein does have an effect on the barrier for translocation, which is larger by about 10 kJ/mol than in absence of calcein. Yet this effect does not depend on the sign of the charge of the NP, as shown in Fig. 4b. We thus conclude that, while calcein might have a minor effect on slowing down the kinetics of the NP-membrane interaction, it does not induce any preferential interaction with either of the oppositely charged Au NPs.

## Conclusions

We have presented the results of a combined experimental and computational study of the interaction between charged monolayer-protected Au NPs and zwitterionic POPC bilayers. We have compared the effect of anionic NP− and cationic NP+ with similar size distributions, ligand shell compositions, and Z-potentials. In both cases, the NP core diameter distribution is peaked at 3–4 nm, a size that allows for passive membrane permeation^13,31–33^. Ligands used in experiments and simulations are the same: the MUS:OT mixture for NP− and the TMA:OT mixture for NP+. By means of QCM-D investigation, we demonstrated that both NP− and NP+ do interact with the free-standing membrane of zwitterionic POPC vesicles, leading to a similar and stable NP mass uptake inside the bilayer. With leakage assays on calcein-loaded POPC liposomes, separately incubated with NP− or NP+, we further showed that both Au NPs do not induce a significative dye release when penetrating inside the bilayer. This result clearly indicates that membrane integrity is not altered by the interaction with our thiol-protected NP− and NP+. Our fluorescence results agree with the data reported by Liu et al.^57^, showing that Au NPs functionalized by strong capping ligands such as the thiols, in their case mercaptopropionic acid and glutathione, do not cause leakage in calcein-loaded phosphocholine liposomes.

The experimental results of this study are corroborated by molecular dynamics simulations. The reliability of the coarse-grained model we use to look at the interaction between model Au NPs and the POPC bilayer has been thoroughly discussed in our previous works^40,54,58^. Our coarse-grained molecular dynamics simulations predict that, at fixed surface charge density, NP− and NP+ should interact with POPC membranes in a very similar way. Moreover, in biased simulations with and without calcein, we see the translocation of water beads through the bilayer during the NP-membrane interaction. Nevertheless, in any of these simulations we observe the translocation of calcein molecules. Despite previous atomistic simulations showed a transient water pore formation during the NP-membrane interaction^54^, this pore is extremely small (in the narrowest region it involves a couple of water beads) and remains open for few ns, presumably too short a time interval to allow for the leak of calcein molecules. These observations can be used as further evidence of the absence of calcein leakage during the NP-membrane interaction, for both NP− and NP+.

These results are in line with the increasingly large body of computational literature covering the interaction of charged molecules with neutral lipid bilayers^59–62^. The embedding of both charged NPs in the membrane core alters in a transient way the membrane integrity, a mechanism of interaction that resembles that of charged amino acids^63^ and monoatomic positive and negative ions^64^. The free energy barriers we calculated for the translocation of the anionic or cationic ligands are also in reasonable agreement with those reported, by all-atom simulations, for charged amino acids^63^ and Na^+^ and Cl^−^ ions^64^.

Our results suggest that, when looking at the interaction between neutral lipid membranes and sub-6 nm anionic or cationic Au NPs having similar core size distribution and ligand shell composition, the sign of the NP charge is not determinant in shaping the NP-membrane interaction. In particular, the opposite sign of the surface charge does not lead to a different effect of NP penetration inside the zwitterionic bilayer. Other factors, such as the shape and size of the NP core, or ligand surface density, length, and surface patterning, are likely to have a larger impact on the formation mechanism of the NP-membrane complex.

## Methods

Experimental error bars reported in the paper refer to standard errors; when standard deviation (σ) is used, it is explicitly indicated.

### Materials for NP and liposome sample preparation

All the materials used for sample preparations are reported in the “Supplementary Information”.

### NP sample preparation

Anionic Au NPs (NP−) were synthesized as described by Canepa et al.^46^; the same one-phase procedure was followed to obtain cationic Au NPs (NP+) (see “Supplementary Information” and Supplementary Figure S8). For NP+, the (11-mercaptoundecyl)-*N*,*N*,*N*-trimethylammonium (TMA) ligand was used instead of MUS. Final NP powders were dispersed in water before use and characterization. Size distributions were measured by TEM and DLS analyses. Z-potential measurements were performed both in water and in the experimental buffer. ^1^H NMR analysis was performed before and after decomposition of the gold core to check for the presence of unwashed excess ligand and to determine the ligand shell composition^65^. Prior to use, NP dispersions were filtered using a 20 nm pore size filter (Anotop 10, Whatman). Concentrations of filtered dispersions were determined by absorption measurements, using a Jasco V-530 spectrophotometer.

### Liposome sample preparation

For leakage assays, sonicated and extruded vesicles were loaded with a buffered self-quenched calcein solution (175 mM, pH 7.4) and separated from non-encapsulated dye using the minicolumn centrifugation technique^66,67^. Lipid concentration after filtration was determined by ^1^H NMR following the procedure of Hein et al.^68^ For QCM-D measurements, sonicated liposomes were prepared in the calcein-free buffer. Liposomes were prepared fresh before experiments and characterized by DLS and Z-potential measurements. Preparation protocols are reported in detail in the “Supplementary Information”.

### Electron microscopy characterization

Bright-field transmission electron microscopy (BF-TEM) acquisitions allowed the investigation of morphology and size of NP− and NP+. We used a Tecnai G2 F20 TWIN TMP TEM, operated at 200 kV. Results and information on sample preparation are reported in the “Supplementary Information” (Supplementary Table S1 and Supplementary Figure S1).

### DLS and Z-potential characterization

For both hydrodynamic size and Z-potential measurements, we used a Malvern Zetasizer Nano ZS instrument (scattered light collected in backscattering at 173°). Sample preparations and data analysis are detailed in the “Supplementary Information” (Supplementary Table S1).

### ^1^H NMR characterization

All NMR spectra were recorded with a Varian Mercury Plus 300 (300 MHz for ^1^H) spectrometer equipped with ATB broadband probe at 27 °C using as internal standard tetramethylsilane (TMS, 0.00 ppm) for NP samples and 3-(trimethylsilyl)propionic-2,2,3,3-*d*_*4*_ acid sodium salt (TMSP, 0.00 ppm) for liposome samples. In the last case, NMR was used to quantify the phospholipid concentration after the minicolumn filtration (see the “Liposome sample preparation” section)^68^. Results and information on sample preparations are reported in the “Supplementary Information” (Supplementary Table S1 and Supplementary Figure S2).

### QCM-D experiments

QCM-D measurements were performed with a QCM-Z500 microbalance (Biolin Scientific) equipped with a thermostated flow chamber. SiO_2_ coated gold sensors (resonance frequency 5 MHz) were used. Before usage, the sensors were subjected to UV/Ozone for 10 min. The higher harmonics 3rd–11th (overtones) were recorded every 1 s during all experiments. Before starting the measurement, the chamber and the access tubing to the chamber (pre-chamber) were filled with the buffer and let to equilibrate at 22 °C until the frequencies of all overtones were stable. For bilayer formation, a concentrated POPC vesicle suspension (3 mg/mL) was diluted with a 100 mM NaCl, 2 mM histidine, 2 mM TES, 0.1 mM EDTA buffer (adjusted to pH 7.4) to reach a final concentration of 0.25 mg/mL. Filtered NP dispersions (25 μg NP) were added to POPC vesicles at a NP/lipid mass ratio, *R*_*m*_ = *m*_NP_/*m*_lip_, of 0.05 and incubated for about 4 h at room temperature, in absence of stirring, before measurements. In the case of NP+, the ionic strength of the buffer was changed after POPC/NP incubation to 200 mM NaCl, to allow vesicle fusion on the sensor^69^. After 600 s equilibration at 22 °C in the pre-chamber, each POPC/NP dispersion (2 mL) was injected into the QCM chamber. All overtones (3rd–11th) were recorded until the supported lipid bilayer formation was observed. The buffer was then exchanged to remove vesicle excess from the chamber. No difference in the frequency signal was recorded before and after rinsing (Fig. 1b). Data were interpreted in the assumption of rigid film formation; in this case, the Sauerbrey equation describes the relationship between the normalized frequency shift (∆*f*/*n*) and change of mass^70^:

1 $$ \Delta m = - C\frac{\Delta f}{n} $$

where ∆*m* is the mass per unit area that is adsorbed on the sensor, *C* is the coefficient that describes the sensitivity of the instrument to changes in mass (*C* ≈ 17.8 ng/(cm^2^ Hz), for a quartz crystal oscillating at 5 MHz); ∆*f* = *f* − *f*_*0*_ is the frequency shift, and *n* is the overtone number. In Fig. 1b, the third overtone was considered, since it is the most stable among the overtones that were investigated.

### Leakage assays

NP-induced vesicle leakage was tested by means of calcein release^71^. Calcein is a membrane-impermeable fluorescent probe, self-quenched at high concentration. It is commonly used for leakage assays involving Au NPs to probe the membrane integrity during the NP-membrane interaction^25,57^. Measurements were performed at 25 °C in a quartz cuvette (2.4 mL) at a lipid concentration of 0.035 mM, in a 100 mM NaCl, 2 mM histidine, 2 mM TES, 0.1 mM EDTA buffer, using a Fluorolog spectrofluorometer (Horiba Jobin-Ivon). Throughout the whole experiment, calcein fluorescence was monitored as a function of time (λ_ex_ = 490 nm, λ_em_ = 520 nm). To ensure the homogeneity of the system before and after NP addition, the sample was continuously stirred with a magnetic bar. We observed that the introduction of the stirring bar in the cuvette caused a transient, reproducible fluorescence increase that reached a plateau after 30 min, as shown in Fig. 2b. This behavior was absent if fluorescence was recorded for the same length of time in the absence of the magnetic bar. We interpreted it as due to the adsorption of lipids by the hydrophobic Teflon surface of the magnetic bar and by the hydrophilic walls of the quartz cuvette, facilitated by stirring and resulting in the disruption of some vesicles with subsequent release of calcein. Similar behavior has already been observed for liposomes of different composition and the extent of release was shown to depend on the lipid mixture and the cuvette material^72^. Therefore, to avoid artifacts, NPs were always added to the POPC vesicle suspension 30 min after introducing the stirrer in the cuvette. In particular, filtered NP dispersions were added to POPC liposomes at a NP/lipid mass ratio, *R*_*m*,_ of 0.03 and 0.05. We chose to use such mass ratios (excluding higher values) as further precaution (in addition to NP Z-potential in buffer) to limit as much as possible NP aggregation before interaction with vesicles. These NP/lipid mass ratios are slightly higher than those used in the literature for similar experiments with Au NPs^73^. Besides, they correspond to a sufficiently high NP uptake measured by QCM as demonstrated in this investigation (Fig. 1b). Therefore, these R_m_ values represent a good compromise to study the effect of NP uptake on the bilayer integrity of calcein-loaded vesicles and to limit undesired NP aggregation in solution. The NP volumes added to POPC liposomes were in the range between 14.6 and 124 μL (Fig. 2d), depending on the concentration of NP aqueous dispersions after filtration. In a typical content release assay, fluorescence data are expressed as:

2 $$Leakage\;\%\;\left(t\right)= \frac{F\left(t\right)-{F}_{0}}{{F}_{max}-{F}_{0}} \%$$

where *F(t)* is the time-dependent fluorescence, *F*_*0*_ the mean fluorescence level immediately before NP addition, and *F*_*max*_ the maximum fluorescence (see Fig. 2b). *F*_*max*_ is determined as the mean fluorescence level immediately after the addition of 0.5% (w/v) sodium cholate to the sample at the end of the experiment to cause the complete leakage of the dye. The data acquisition rate was 1 point per second.

### Coarse-grained models

The model of our Au NPs, described and validated in our earlier work^40,54^, comprises an atomistic description of the Au core (2 nm diameter) with a coarse-grained representation of the ligand shell. The coarse-grained model is based on the polarizable water Martini force field^52,53^. Anionic ligands are described by a chain of 3 hydrophobic C_1_ beads and one negatively charged terminal Q_da_ bead. The model of the cationic ligand is identical except for the terminal bead representing the trimethylammonium ion, which is described by a positively charged Q_0_ bead. The NPs are covered by 30 hydrophobic ligands and 30 charged ligands with a random grafting on the Au core. The parameterization of the Martini coarse-grained model of the calcein dye is described in Ref.^56^.

### Simulation set-up

Unbiased and metadynamics^55^ simulations without calcein were run following the simulation set-up described in our previous work^40,54^. In short, one NP was placed in contact with the POPC bilayer (see Fig. 3); the bilayer is composed of 512 POPC lipids, and the simulation box size is 13 × 13 × 18 nm^3^. A solution of 150 mM NaCl was used, plus 30 counter ions to balance the NP charge. In the simulations with calcein, the box is composed of two aqueous compartments separated by two identical POPC membranes, composed of 512 lipids each. In one compartment, there is calcein (175 mM), together with a physiological solution of NaCl (150 mM) and Na^+^ counter ions. In the other compartment, there are the NP in contact with the membrane, the same NaCl physiological solution, and the NP counter ions. We performed simulations in the isothermal-isobaric (NPT) ensemble. We used the velocity-rescale thermostat to set the temperature to 310 K. The pressure was kept constant to 1 bar with the Berendsen and the Parrinello-Rahman algorithms for the equilibration and production run, respectively. The long-range contribution of electrostatics was included with the PME method and a Fourier grid spacing of 0.12 nm is used. We used a time step of 20 fs. All simulations were performed with Gromacs 2016^74^ and Plumed^75^. Metadynamics^55^ simulations were performed following the set-up described in our previous work^54^. Briefly, the collective variable is the distance between one charged terminal group and the membrane COM along the membrane normal. The gaussian height and width are 2.48 kJ/mol and 0.06 nm, respectively, and the deposition time is 1 ns.

### Contact analysis

The number of water contacts with the biased charged terminal group was obtained with the Gromacs *mindist* tool with a cut-off distance of 0.6 nm.

## Supplementary Information

Supplementary Information.

## Data Availability

The dataset used and/or analyzed during the current study are available from the corresponding author upon reasonable request after publication. The topology files and input configurations for all computer simulations performed in this study are available free of charge in our online repository: https://bitbucket.org/biomembnp/biomembnp.
