# Supplementary material for: Non-disruptive uptake of anionic and cationic gold nanoparticles in neutral zwitterionic membranes
Source: Sci Rep. 2021 Jan 13;11:1256. doi: 10.1038/s41598-020-80953-3 (PMC7807088; doi:10.1038/s41598-020-80953-3)
Supplement: Supplementary file 1 — Supplementary Information. [file 41598_2020_80953_MOESM1_ESM.pdf]

Supplementary Information

Non-disruptive uptake of anionic and cationic gold nanoparticles in neutral zwitterionic membranes

Ester Canepa[1,+], Sebastian Salassi[2,+], Federica Simonelli[2,+], Riccardo Ferrando[2], Ranieri Rolandi[2], Chiara Lambruschini[1], Fabio Canepa[1], Silvia Dante[3], Annalisa Relini[2], and Giulia Rossi[2,*]

[1]Department of Chemistry and Industrial Chemistry, University of Genoa, 16146 Genoa, Italy

[2]Department of Physics, University of Genoa, 16146 Genoa, Italy

[3]Materials Characterization Facility, Italian Institute of Technology, 16163 Genoa, Italy

*relini@fisica.unige.it; rossig@fisica.unige.it

[+] these authors contributed equally to this work

Experimental characterization of NP–, NP+ and POPC liposomes

	Core size (nm)	Hydrodynamic size (nm)	Ligand shell composition (%)	Z-potential in water (mV)	Z-potential in buffer (mV)
**NP–**	2.7 ± 0.8 (σ)	6.5 ± 0.2	80 ± 8 anionic ligand	–48 ± 4	–31 ± 3
**NP+**	4.5 ± 1.1 (σ)	7.7 ± 1.4	72 ± 11 cationic ligand	+38 ± 3	+25 ± 2
**POPC liposomes**	-	23 ± 2 (sonicated)<br>105 ± 6 (extruded)	-	-	–5.5 ± 0.4

Supplementary Table S1. Characterization results of NP–, NP+ and POPC liposomes. All the uncertainties on mean values were obtained using Student's statistics, assuming a confidence level of 95%. Standard deviation (σ) is reported only for NP core size distributions (see below for more information). For both hydrodynamic size and Z-potential analyses, at least 9 measurements were performed; hydrodynamic diameters were evaluated from the average diameters obtained from number distributions. In the case of ligand shell composition, three NP spectra were acquired.

For liposome characterization, all analyses were performed in the 100 mM NaCl, 2 mM histidine, 2 mM TES, 0.1 mM EDTA buffer (pH 7.4) used for experiments.

For NP characterization, NP powders were dispersed in water and, when specified, diluted in buffer. Only for NP+, aqueous dispersions were further centrifuged before DLS, Z-potential, and NMR characterizations (see the NP sample preparation section). For DLS size analysis in water, measurements were acquired at $2{\cdot}10^{-2}$ mg/mL NP concentration (ca. ten-fold higher than those corresponding to R_m=0.03 and 0.05) since lower values did not provide good quality reports. In the case of DLS and Z-potential measurements, results refer to NP dispersions freshly filtered immediately before experiments (20 nm pore size, Anotop 10, Whatman). All NP dispersions were mildly sonicated before analyses.

BF-TEM analysis: NP core size distribution

Few drops of each NP water dispersion were laid onto an ultrathin carbon-coated Cu grid. In Supplementary Figure S1, representative BF-TEM images of NP– and NP+ are presented, together with histograms of the experimental size distribution. Statistical analysis was calculated by assuming spherical morphology and by counting at least 300 particles with ImageJ software.

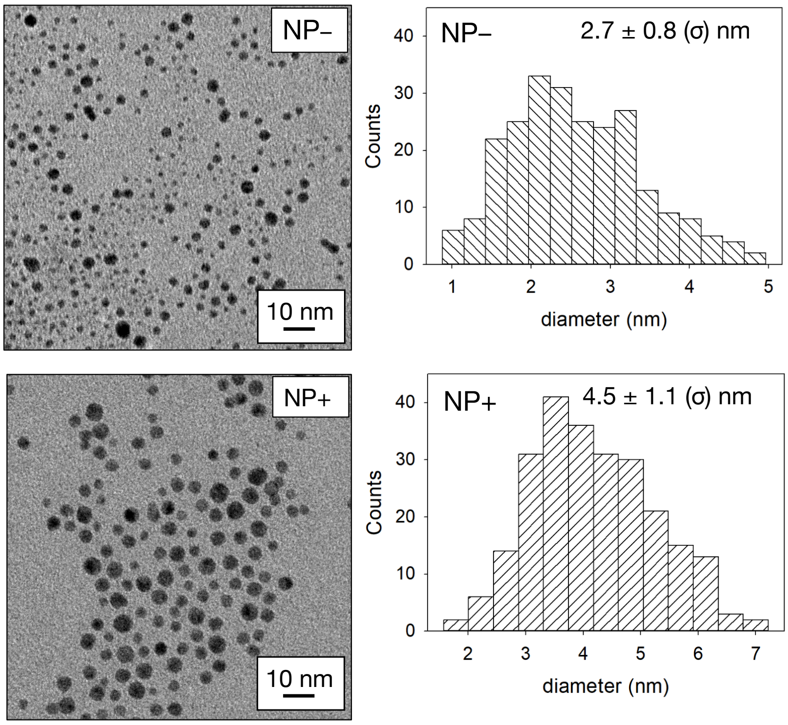

Supplementary Figure S1. BF-TEM images of NP– and NP+. Experimental size distributions are shown aside; mean diameters and standard deviations (σ) are reported above each histogram.

^{1}H NMR analysis: purity and ligand-coating composition of NP– and NP+

NP purity assessment. ^{1}H NMR was firstly used to assess the presence of unbound ligands in NP– and NP+ samples. Charged NPs (~ 5 mg) were dispersed in MeOH-d_4 (600 μL), the sample was sonicated for 30 min and ^{1}H NMR spectrum was acquired. In both cases, the absence of clear and sharp peaks indicated that no unbound ligands were present in our NP samples.

NP ligand-coating composition analysis. ^{1}H NMR was also used to assess the Au NP ligand shell composition (i.e. the MUS % and the TMA % in NP– and NP+, respectively) after decomposition of the gold core[4]. In the case of NP–, the procedure for the determination of MUS% and the NMR spectrum are reported in Canepa *et al.*[1]. The same procedure described for NP– was used to characterize TMA% in NP+. Only minor modifications were added to the sample preparation. NP+ (~ 5 mg) were dispersed in water (2 mL) and mildly centrifuged (10 min at 11900 X *g*), only the supernatant was kept. This procedure was repeated, and the collected aqueous suspensions were evaporated to dryness. The residue was taken up in DMSO-d_6 (600 μL), I_2 (1.6 mg) was added and the mixture was sonicated for 30 min to etch the gold core. The orange solution was transferred into 5 mm NMR tube and analyzed as for NP–. NP+ spectrum is reported in Supplementary Figure S2. NMR results are summarized in Supplementary Table S1.

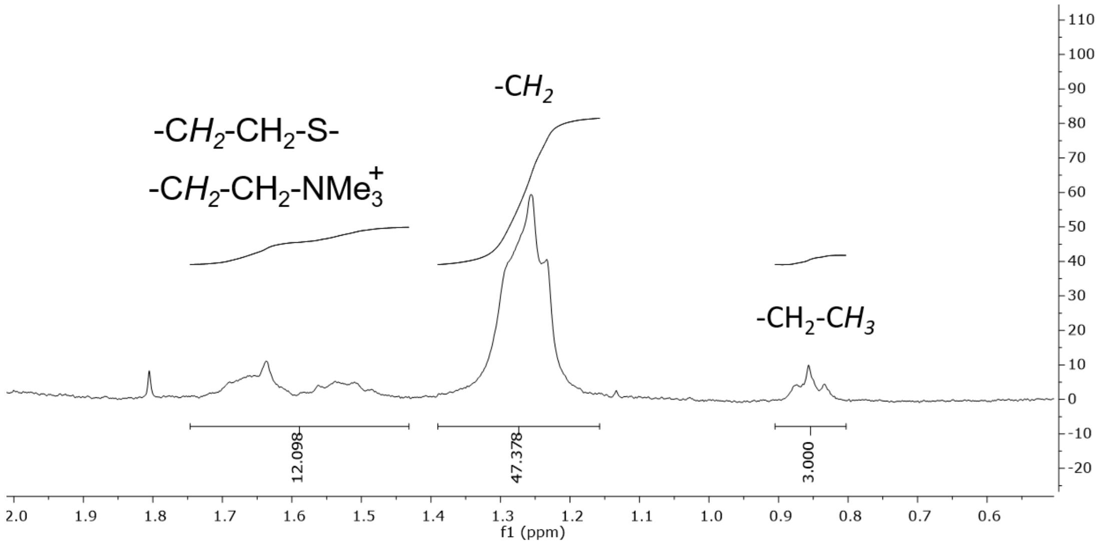

Supplementary Figure S2. Expansion of ^{1}H NMR spectrum of NP+ after centrifugation and iodine etching in DMSO-d_6.

Experimental results

QCM-D investigation: dissipation changes

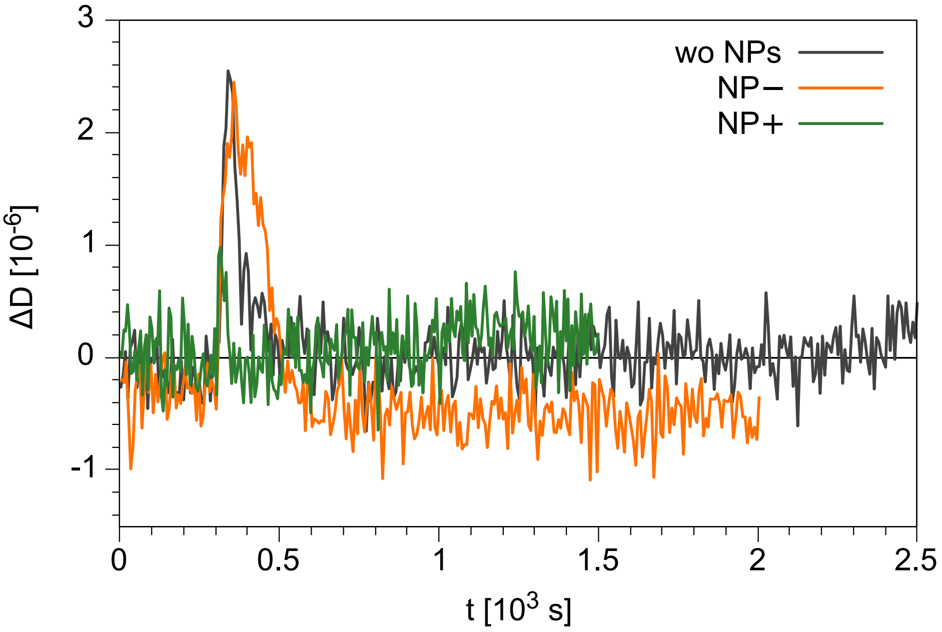

Supplementary Figure S3. Dissipation changes (ΔD) recorded after the injection (t = 300 s) in the QCM-D chamber of POPC vesicles (black curve), POPC vesicles incubated with NP– (POPC/NP–, orange curve) and POPC vesicles incubated with NP+ (POPC/NP+, green curve). These traces were recorded simultaneously with the Δf curves shown in Figure 1b: ΔD follows the same kinetics of SLB formation shown by Δf (i.e. its maximum corresponds to the Δf minimum). In the case of POPC and POPC/NP–, the dissipation variations are comparable. In the case of POPC/NP+, only a small increase right after NP injection was recorded, followed by the instantaneous formation of the SLB which is evidenced by the ΔD decrease to zero.

Content release assays on POPC liposomes

To understand whether the origin of minor fluctuations shown in Figure 2d of the main text could be due to the addition of charged NPs, we performed control leakage experiments adding similar volumes of water aliquots (without NPs). From Supplementary Figure S4a, it is evident that the effect of the water alone is comparable with those of both NP– and NP+.

Additional leakage assays were performed on larger POPC vesicles prepared by extrusion, as reported in Supplementary Figure S4b. These experiments show that the increase in vesicle diameter from 23 to 105 nm does not change the effect of the NP-membrane interaction (see Supplementary Table S1 for results of liposome size characterization).

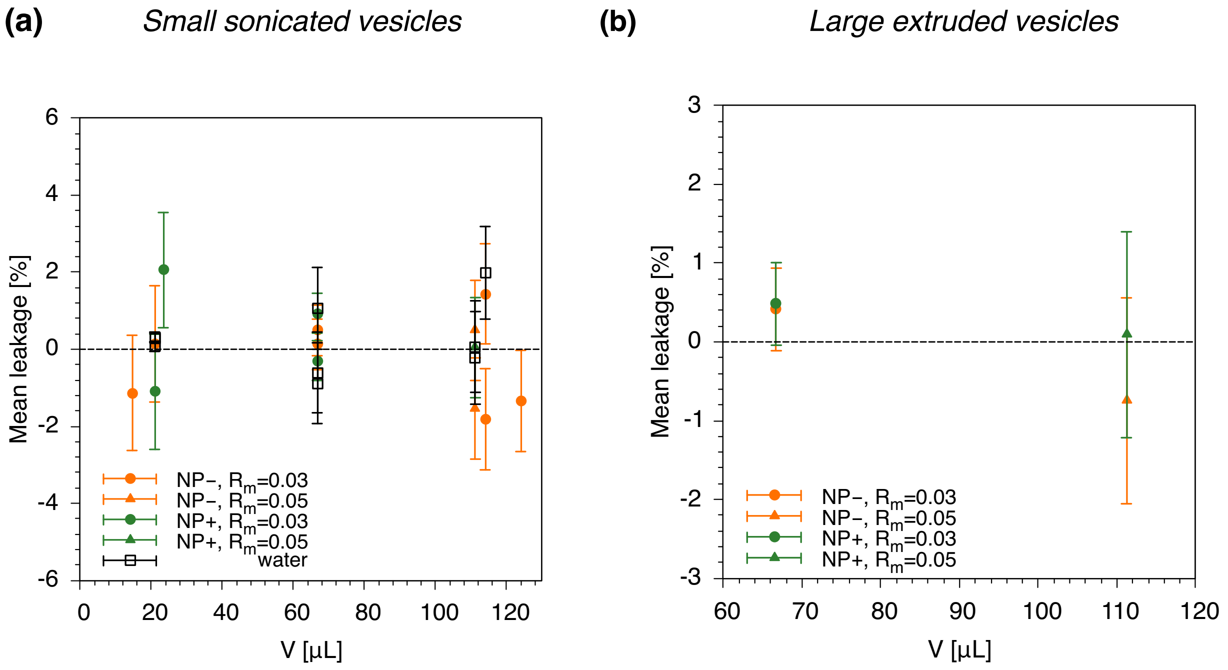

Supplementary Figure S4. Summary of all content release assays performed on POPC liposomes. (**a**) Comparison of the effect of the addition of charged Au NPs (at different R_m) and water aliquots on small (sonicated) POPC liposomes. (**b**) Effect of the addition of charged Au NPs (at different R_m) on large (extruded) POPC liposomes. NP– (orange) and NP+ (green) at $R_m = 0.03$ (●) and 0.05 (▲); water aliquots (□). In both plots, the normalized fluorescence intensity of calcein (i.e. mean leakage %), calculated over the last 10 min before the addition of the detergent, is reported as a function of the added volume.

Computer simulations

We monitored the area per lipid of a 512 POPC lipid bilayer, in presence of 175 mM calcein in the water phase (and no NP) and found that both the area per lipid and the membrane compressibility are the same as without calcein.

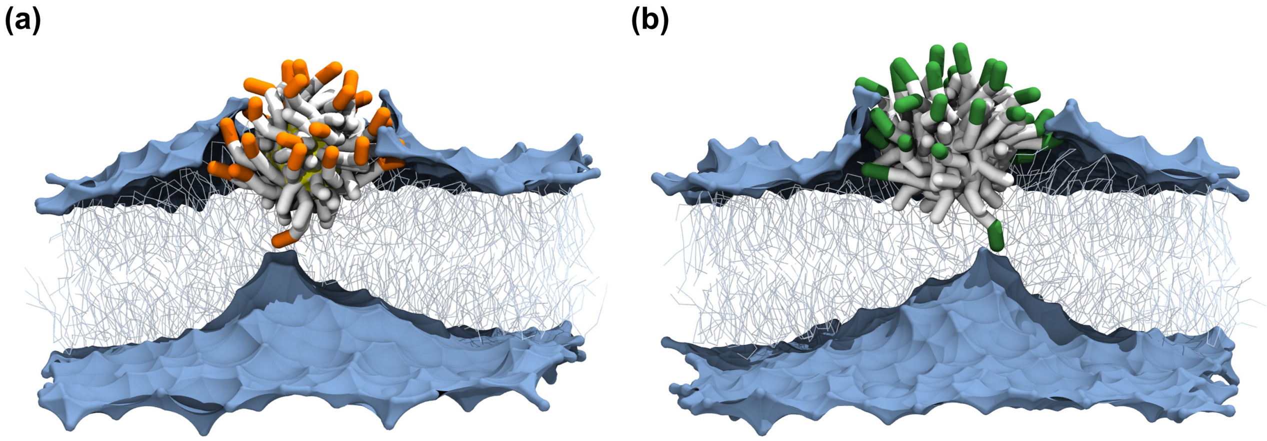

Supplementary Figure S5. Membrane deformations caused by anchoring of one charged ligand, as observed during metadynamics runs for (**a**) NP– and (**b**) NP+ inserted in a POPC membrane. Anionic ligand beads in orange, cationic ligand beads in green, hydrophobic ligand beads in white, lipid heads in blue (surface representation), and lipid tails in light-gray sticks. Here and in the following figures, water is not shown.

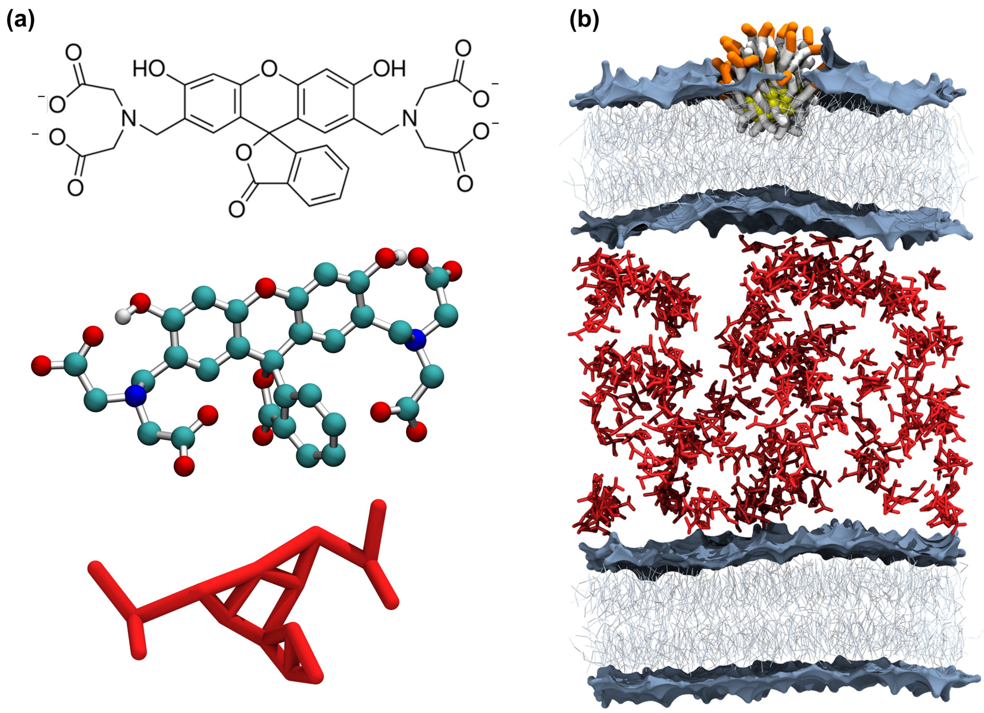

Supplementary Figure S6. Model of the calcein dye**.** (**a**) Atomistic and coarse-grained structure of calcein. (**b**) Set-up of the metadynamics simulations with calcein. The box contains two membranes and two separate water chambers. One of the water chambers contains calcein molecules (red) at 175 mM concentration (the same as for leakage experiments).

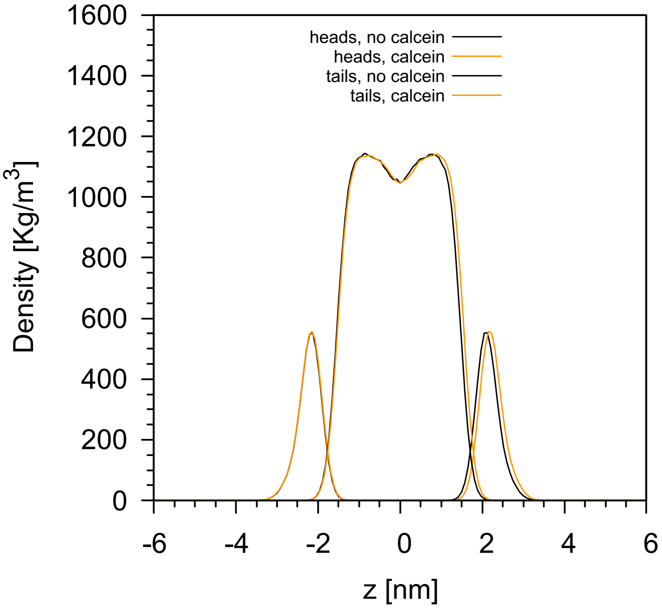

Supplementary Figure S7. Density profiles of lipids in the presence (orange) and absence (black) of calcein.

NP sample preparation

Materials for NP sample preparation. Hydrogen tetrachloroaurate (III) hydrate ($HAuCl_4 \cdot 3H_2O$, 99.999 % trace metal basis), 1-octanethiol (OT, ≥ 98.5 %), (11-mercaptoundecyl)-*N*,*N*,*N*-trimethylammonium bromide (TMA, 98 %), sodium borohydride (fine granular, ≥ 96 %), ethanol absolute (> 99.8 %), anhydrous methanol (99.8 %), methanol (≥ 99.9 %), acetone (≥ 99.9 %) and 2-propanol (≥ 99.5 %) were purchased from Sigma Aldrich and used without further purification. The anionic ligand 11-mercapto-1-undecanesulfonate (MUS, 93%) was prepared in house[1]. All aqueous solutions were prepared using water purified with a Milli-Q ultrapure water system (MilliPore).

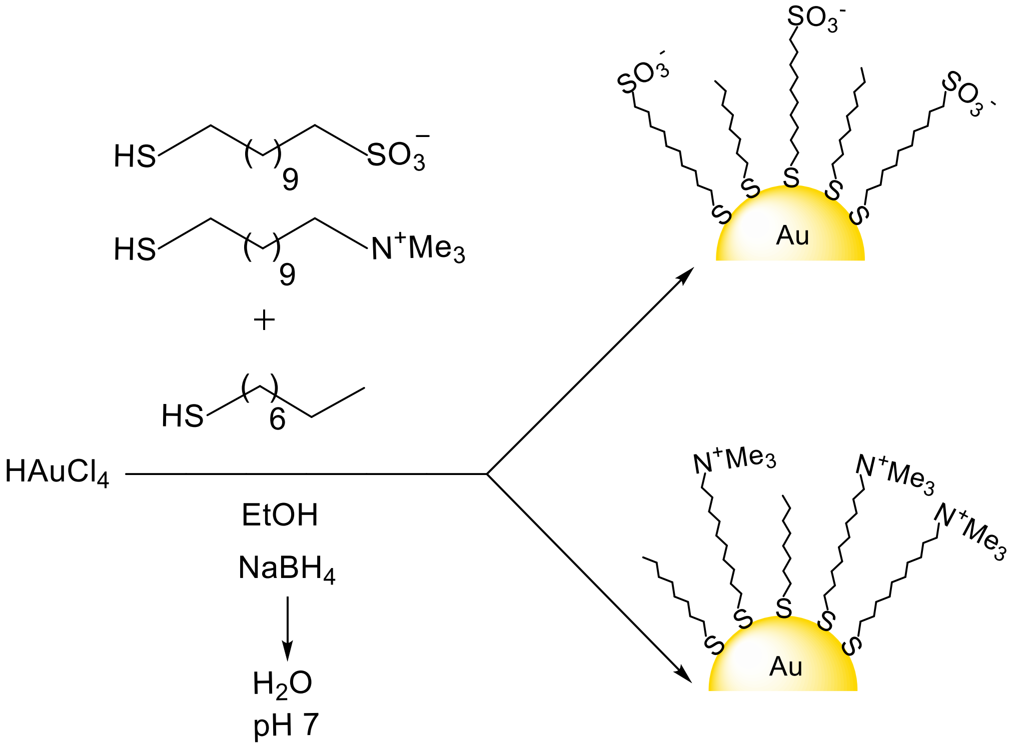

Supplementary Figure S8. Schematic drawing illustrating the one-phase synthesis of anionic and cationic Au NPs. An amphiphilic thiol mixture composed of the apolar 1-octanethiol (OT) and either the anionic 11-mercapto-1-undecanesulfonate (MUS) (sodium salt) or the cationic (11-mercaptoundecyl)-*N*,*N*,*N*-trimethylammonium (TMA) (bromide salt) was used to obtain anionic MUS:OT Au NPs (NP–) and cationic TMA:OT Au NPs (NP+), respectively.

We obtained NP– and NP+ with the one-phase method shown in Supplementary Figure S8. We used different optimized washing protocols for the two kinds of NPs, consisting of repeated overnight precipitations at -21°C in ethanol, methanol, and acetone for NP– and in ethanol and 2-propanol for NP+. Since NP+ showed slightly less colloidal stability over time than NP–, NP+ water dispersions were mildly centrifuged (10 min at 11900 X *g*) to remove the minority of unstable NPs and only the stable colored supernatant was kept for experiments and DLS, Z-potential, and NMR characterizations.

Liposomes sample preparation

Materials for liposome sample preparation. POPC (lyophilized powder) was purchased from Avanti Polar Lipids (Alabaster, AL), calcein (Merck, ≥ 96 %) was purchased from VWR International PBI. L-Histidine (≥ 99 %), 2-[tris(hydroxymethyl)methylamino]-1-ethanesulfonic acid (TES, ≥ 99 %), ethylenediaminetetraacetic acid disodium salt (EDTA, 99.0 - 101.0 %), sodium chloride (≥ 99.5 %) and chloroform (≥ 99.5 %) were purchased from Sigma Aldrich and used without further purification.

Lyophilized POPC was weighed in a glass vial, dissolved in chloroform, and divided into aliquots. Lipid solutions were gently dried under a stream of nitrogen to form homogeneous films, which were put under vacuum for 24 h in a desiccator to remove residual solvent. For membrane leakage assays, POPC films were hydrated at a lipid concentration of 1 mg/mL in a 175 mM calcein, 2 mM histidine, 2 mM TES buffer solution (adjusted to pH 7.4 using NaOH and HCl). This calcein concentration is self-quenched. Hydration was made easier by employing a bath-type sonicator for 10 min to form multilamellar vesicles. The multilamellar dispersion was then sonicated with a probe-type sonicator under nitrogen at 25 °C for 15 min to obtain small unilamellar liposomes. To remove large lipid aggregates and titanium impurities, the liposomal preparation was centrifuged for 10 min at 11500 X *g*. Additional larger calcein-loaded vesicles were prepared using the Avanti Mini-Extruder (Avanti Polar Lipids). In this case, after the hydration step in the bath-type sonicator, the lipid suspension was extruded 11 times at room temperature with a 100 nm pore size polycarbonate membrane. Sonicated and extruded calcein-loaded vesicles were separated from the non-encapsulated dye by gel filtration using the minicolumn centrifugation technique[2,3]. The minicolumns (1.0 cm x 10 cm; Pierce) were filled with Sephadex G-50 swollen in a 2 mM histidine, 2 mM TES, 100 mM NaCl, 1.0 mM EDTA buffer solution (adjusted to pH 7.4) and centrifuged twice for 1 min at 3000 X *g* to remove the excess buffer from the gel filtration medium. The vesicle preparation was then divided into aliquots of 100 μL per column and filtered by centrifugation at 3500 X *g* for 1 min. The lipid concentration after vesicle filtration was determined by ^{1}H NMR, following the procedure described by Hein et al.[4] For QCM-D measurements, sonicated calcein-free POPC liposomes (3 mg/mL) were prepared using the 2 mM histidine, 2 mM TES, 100 mM NaCl, 1.0 mM EDTA buffer instead of the self-quenched calcein solution.

<u>*^{1}H NMR analysis: POPC liposome concentration after filtration*</u>

The quantification of the phospholipid content in liposome samples after the minicolumn filtration (see the liposome preparation section) was carried out following the procedure reported by Hein et al.[4] with minor modifications. The analysis was performed using the 3-(trimethylsilyl)propionic-2,2,3,3-d_4 acid sodium salt (TMSP, 0.00 ppm) as internal standard. 200 μL of liposome solution was

directly mixed with 430 μL CD_3OD, 100 μL $CDCl_3$, and 15 μL 5.9 mM TMSP in D_2O in an NMR tube. After mixing, a clear and homogeneous solution was obtained and directly analyzed. After integration, the concentration of phospholipid was calculated and resulted to be 1.15 mM (0.9 mg/ml) of POPC. This concentration was diluted before the following leakage assays.

Details of NMR liposome analysis. (H)PRESAT parameters: from –2.0 to 14.0 ppm spectral window, presaturation of H_2O peak (δ 4.6 ppm), 256 scans, 3.0 s relaxation delay. Post-acquisition processing was performed with MestReNova (Mestrelab research v. 11.0): manual phase correction, 264 k zero filling, 3rd order polynomial baseline correction, apodization LB = 1.0 Hz and manual integration. Peaks assignments were made on the basis of chemical shifts. The following equation was employed to calculate the concentration of phospholipid:

$$C_{PL} = \frac{3C_{Std}V_{Std}I_{PL}}{2V_{PL}I_{Std}}$$

Where C_{PL} is the final concentration of phospholipid (mM), C_{Std} is the concentration of TMSP (mM), V_{PL} is the volume of phospholipid (μL), V_{Std} is the volume of TMSP (μL), I_{PL} is the integral value of terminal CH_3 of phospholipid (δ 0.90 ppm) and I_{Std} is the integral value of TMSP (δ 0.00 ppm).

References

1. Canepa, E. *et al.* Amphiphilic Gold Nanoparticles Perturb Phase Separation in Multidomain Lipid Membranes. *Nanoscale* (2020) doi:10.1039/D0NR05366J.
2. Relini, A. *et al.* Effect of physical constraints on the mechanisms of membrane fusion: Bolaform lipid vesicles as model systems. *Biophys. J.* **71**, 1789–1795 (1996).
3. Lasch, J., Weissig, W. & Brandl, M. Preparation of Liposomes. in *Liposomes: A Practical Approach* (ed. Torchilin, Vladimir and Weissig, V.) 33–104 (Oxford University Press, 2003).
4. Hein, R., Uzundal, C. B. & Hennig, A. Simple and rapid quantification of phospholipids for supramolecular membrane transport assays. *Org. Biomol. Chem.* **14**, 2182–2185 (2016).
